# Evaluation of Polmacoxib 2 mg for the Management of Hip and Knee Osteoarthritis: A Prospective, Single-Arm, Multicenter, Open-Label Study

**DOI:** 10.7759/cureus.94838

**Published:** 2025-10-18

**Authors:** Rakesh Verma, Rajiv Gupta, Pravin Waghmare, Sanjeev Kumar Kare, Ajit K Kar, Urvi K Mistry, Milind Bhole, Swapnil Deshpande, Sujay Patil

**Affiliations:** 1 Orthopedics, Jawaharlal Nehru Medical College, Ajmer, Ajmer, IND; 2 Orthopedics, Bhandari Hospital and Research Centre, Jaipur, IND; 3 Orthopedics, Punawale Multispeciality Hospital, Pune, IND; 4 Orthopedics, Government Medical College, Srikakulam, Srikakulam, IND; 5 Orthopedics, Shanti Memorial Hospital, Cuttack, IND; 6 Medicine, Abbott Healthcare Pvt. Ltd., Mumbai, IND; 7 Established Pharmaceuticals Division, Abbott Healthcare Pvt. Ltd., Mumbai, IND; 8 Medical Affairs, Abbott Healthcare Pvt. Ltd., Mumbai, IND; 9 Clinical Research and Development, Abbott Healthcare Pvt. Ltd., Mumbai, IND

**Keywords:** cyclooxygenase 2 inhibitor, osteoarthritis, pain management, polmacoxib, womac index

## Abstract

Purpose: Polmacoxib is a novel cyclooxygenase-2 (COX-2) selective inhibitor with effective analgesic and anti-inflammatory properties and a favorable safety profile. This study evaluated polmacoxib's effectiveness, onset of action, and tolerability in Indian patients with knee or hip osteoarthritis.

Method: This was a prospective, multicenter, single-arm, open-label, observational study among Indian patients with knee/hip osteoarthritis. All patients with knee/hip osteoarthritis received polmacoxib capsule 2 mg orally once daily for six weeks. Patients were followed up for eight weeks (six weeks of treatment + two-week follow-up). Outcomes evaluated were pain, stiffness, and physical function using Western Ontario and McMaster Universities Arthritis Index (WOMAC) (CRD Pune version), severity of pain by Visual Analog Scale (VAS), and time of onset of action (in minutes), post-taking the first dose of polmacoxib 2 mg capsule on Day 1. Safety and compliance were monitored throughout the study period.

Results: In the study, 150 (male: female, 89:61) patients with a mean (standard deviation, or SD) age of 46.6 (9.9) years were enrolled. Statistically and clinically significant improvements were observed in all parameters. The mean (SD) pain score on the WOMAC Index declined significantly by -2.6 (2.1) and -5.9 (3.1) at Weeks 3 and 6, respectively, compared to the baseline score of 11.4 (4.6). Compared to the baseline mean (SD) score of 4.9 (2.1), the stiffness score on the WOMAC Index declined significantly by -1.0 (1.0) at Week 3, and by -2.4 (1.3) at Week 6. Mean (SD) difficulty (in physical function) score on the WOMAC Index declined significantly by -7.8 (5.9) and by -19.8 (10.2) at Weeks 3 and 6 respectively, compared to the baseline score of 41.6 (15.7), leading to a significant decline in total WOMAC Index by -11.4 (7.9) and by -28.1 (13.8) at Weeks 3 and 6 respectively, compared to the baseline score of 57.9 (21.9). The mean (SD) intensity of pain score (VAS) also declined significantly by -1.3 (1.3) and -2.9 (1.9) at Weeks 3 and 6, respectively, compared to the baseline score of 7.5 (0.9). Six (4.0%), 69 (46.0%), 62 (41.3%), and 13 (8.7%) of patients reported the onset of action of the study drug within 15, 30, 45, and 60 minutes of the first dose, respectively. The drug was well tolerated, with only 2% of patients experiencing mild, unrelated adverse events. Tolerability was rated “Excellent to Very Good” in 96.0% of cases by investigators and 89.3% of patients.

Conclusion: Polmacoxib 2 mg demonstrated early onset of symptom relief, significant improvements in clinical symptoms, and an acceptable safety and tolerability profile over a six-week treatment period, suggesting that polmacoxib is a well-tolerated treatment option for osteoarthritis patients necessitating effective and rapid symptom control with minimal systemic side effects.

## Introduction

Osteoarthritis is the most prevalent form of arthritis [1], characterized by progressive joint degeneration accompanied by low-grade inflammation contributing to symptomatology and disease progression. It leads to the breakdown of cartilage and damage to the surrounding bone tissue, resulting in pain, swelling, and reduced mobility [2]. In 2020, around 1 in 13 people worldwide had osteoarthritis, and the numbers are expected to rise sharply by 2050, especially for knee and hip osteoarthritis [1]. The disease primarily affects older adults, with osteoarthritis ranked as the seventh leading cause of disability in those aged 70 and above [1]. High body weight is a key contributor to osteoarthritis risk, and other modifiable risk factors like injuries and occupational hazards remain under-investigated [1]. Thus, with the combination of an aging population and rising obesity, it is predicted that the prevalence of osteoarthritis may increase worldwide [3].

The treatment regimens of osteoarthritis mainly focus on managing pain and improving joint function through lifestyle or therapeutic interventions, due to the lack of a definitive cure [3]. The lifestyle interventions include joint care measures such as avoiding activities that worsen the pain or overload the joint, exercising to improve strength, maintaining a healthy body weight, and incorporating occupational therapy [4-6]. Therapeutic approaches for osteoarthritis involve nonsteroidal anti-inflammatory drugs (NSAIDs) and opioids, but these medications carry a high risk of adverse effects (AEs), such as gastrointestinal (GI) disorders [7-12]. NSAIDs work by inhibiting cyclooxygenase (COX1 and COX2) enzymes that regulate inflammation [3]. While COX enzymes are involved in inflammatory pathways [13], COX-1 also plays a protective role in maintaining the GI mucosa through prostaglandin synthesis [14,15]. Thus, due to nonselective inhibition of both COX-1 and COX-2, traditional NSAIDs reduce inflammation but may also cause GI damage [14]. COX inhibition has been associated with an increased risk of adverse cardiovascular (CV) events as well, such as elevated blood pressure and myocardial infarction [16,17]. To reduce GI complications, COX-2 selective inhibitors (e.g., celecoxib, rofecoxib, etc.) were developed and have shown improved GI safety profiles [18,19]; however, selective COX-2 inhibition does not eliminate the risk of adverse CV events.

Polmacoxib is a new, orally active, dual inhibitor of COX-2 and carbonic anhydrase (CA) enzymes [20]. CA regulates the level of carbon dioxide and bicarbonate in the circulatory system to maintain the body’s pH balance [21]. Therefore, in CV tissues where COX-2 and CA are co-expressed, polmacoxib’s strong affinity binding to the latter may attenuate the inhibitory effect on COX-2 [22], potentially minimizing the adverse CV effects [23]. Although clinical evidence supporting this theory is currently limited but the dual inhibitory action of polmacoxib positions it as a promising candidate for osteoarthritis treatment. Evidence on the safety and efficacy of polmacoxib in osteoarthritis is limited to two studies. A phase III multicenter trial by Lee et al. found polmacoxib to be well tolerated, with efficacy superior to placebo and non-inferior to celecoxib over six weeks, with consistent results during an 18-week extension [3]. Another recent randomized, double-blind study in an Indian cohort also confirmed its non-inferiority to celecoxib in terms of safety and efficacy over six weeks [24]. Therefore, this prospective, multicenter, single-arm, open-label observational study was conducted to complement the existing evidence by evaluating the effectiveness, onset of action, and safety of polmacoxib 2 mg in a real-world Indian adult population with hip or knee osteoarthritis over six weeks, followed by a two-week follow-up.

## Materials and methods

Patient selection

This prospective, multicenter, observational study employed a single-arm, open-label design (CTRI/2024/06/069581; registered on 26 June 2024) that aimed to evaluate the effectiveness, onset of action, and the safety and tolerability of six-week treatment with polmacoxib 2 mg capsules (Abbott Healthcare Private Limited) in the management of hip or knee osteoarthritis in Indian patients. The study was conducted from August to December 2024 at five sites across different geographical locations in India.

The inclusion criteria required patients aged 20-70 years with a clinically confirmed diagnosis of knee/hip osteoarthritis, having chronic pain for three months or longer due to osteoarthritis, baseline (pre-dose) mean Western Ontario and McMaster Universities Arthritis Index (WOMAC) (CRD Pune version) pain score between 4 and 8 in the affected joint, and willing to give written informed consent and adhere to all study-related procedures.

The exclusion criteria included the use of any analgesics other than the study drug or paracetamol within 24 hours before the first dose and throughout the study period; anticoagulant therapy within two weeks prior to enrollment; and use of corticosteroids, herbal or ayurvedic remedies, nutraceuticals, glucosamine, and/or chondroitin sulfate within three months before study entry. Patients were also excluded if they required or expected to require knee or hip arthroplasty within two months of screening, or if any surgery involving the index joint was anticipated during the study. Additional exclusion criteria included known hypersensitivity to NSAIDs; any condition deemed unsuitable by investigators as per prescribing information; a history of nasal polyps, bronchospasm, urticaria, or anaphylaxis; and significant CV conditions such as a history of New York Heart Association stage II-IV congestive heart failure, ischemic heart disease, uncontrolled hypertension, peripheral arterial disease, and/or cerebrovascular disease. Pregnancy, breastfeeding, or plans for conception during the study were excluded, as were active ulcers, GI bleeding, ulcerative colitis, or severe renal, hepatic, or coagulation disorder within six months of the study enrollment. Patients with persistent psychiatric or chronic medical conditions that could interfere with study compliance, unless physically healthy and receiving the specified allowed drugs for at least three months, were excluded, as were those who received corticosteroids, intra-articular steroids, or hyaluronic acid injections within one month prior to screening or chemotherapy within the past five years.

Study design

The total duration of the study was eight weeks (six weeks of treatment + two-week follow-up). Enrolled patients were prescribed the study drug polmacoxib 2 mg capsule, to be taken orally once daily, as per the routine clinical practice and label, for a period of six weeks. Patients were followed up in the clinic at Week 3 (± 1 day) and Week 6 (±1 day). In addition to this, there was one telephonic follow-up for safety assessment at Week 8.

The study was conducted in accordance with good clinical practices (GCP) guidelines and the New Drugs and Clinical Trial Rules, 2019 (India), with a strong emphasis on safeguarding participants’ rights, safety, and well-being. Ethical standards outlined in the Declaration of Helsinki were adhered to throughout the study. The study protocol and informed consent documentation received prior approval from the Institutional Ethics Committee at each participating center before the initiation of the study. Written informed consent for participation and the use of medical data was obtained from all patients before initiating any assessments.

Study endpoints

The effectiveness of the intervention was evaluated based on improvements in clinical symptoms of knee/hip osteoarthritis, as assessed using the WOMAC Index (Modified - CRD Pune Version) [25] and a 10-point Visual Analog Scale (VAS). The WOMAC Index (Modified - CRD Pune Version) [25,26] is an adapted version of the original WOMAC Osteoarthritis Index [27], developed and validated by the Centre for Rheumatic Diseases (CRD), Pune, India, as an outcome measure for arthritis. Formal permission to use the WOMAC index (Modified - CRD Pune Version) in this study was obtained from the copyright holders, CRD Pune, India, following payment of the requisite copyright fee.

The 24-question WOMAC Index (Modified - CRD Pune Version) retains the original three components of the WOMAC Index. While the "Pain" (five questions) and "Stiffness" (two questions) components remain unchanged, the "Physical Function/Difficulty" component (17 questions) has been modified to better reflect Indian customs and daily living habits [25,26]. Each question is scored on a 5-point scale: 0 = None, 1 = Mild, 2 = Moderate, 3 = Severe, and 4 = Extreme, with a total maximum score of 96 (Table 1).

**Table 1:** WOMAC Index (Modified - CRD Pune Version) Permission to use the Western Ontario and McMaster Universities Arthritis Index (WOMAC) (Modified - CRD Pune Version) was obtained from the copyright holder, the Centre for Rheumatic Diseases (CRD), Pune, India. The instrument was sourced from official documentation provided by CRD and utilized in this study with appropriate authorization.

	None	Mild	Moderate	Severe	Extreme
How Much Pain Do You Have? (Circle one number for each activity)					
In Walking on flat surface	0	1	2	3	4
Going up or down stairs	0	1	2	3	4
At night while in bed	0	1	2	3	4
Sitting or lying	0	1	2	3	4
Standing upright	0	1	2	3	4
Total Pain Score (Sum of Score of items 1 to 5)					
How Much Is Your Stiffness? (Circle one number for each activity)					
After first wakening in the morning	0	1	2	3	4
After sitting, lying or resting later in the day	0	1	2	3	4
Total Stiffness Score (Sum of Score of items 1 and 2)					
How Much Difficulty Do You Have? (Circle one number for each activity)					
Descending stairs	0	1	2	3	4
Ascending stairs	0	1	2	3	4
Standing up from a chair	0	1	2	3	4
While Standing	0	1	2	3	4
Bending to floor (to pick up object)	0	1	2	3	4
Walking on a flat ground	0	1	2	3	4
Getting in/out of auto rickshaw/ bus/ car	0	1	2	3	4
Going shopping	0	1	2	3	4
On rising from bed	0	1	2	3	4
While lying in bed	0	1	2	3	4
While sitting on chair	0	1	2	3	4
Going on/off - Indian/Western	0	1	2	3	4
Doing heavy domestic duties (moving heavy boxes, scrubbing floor, lifting shopping bags)	0	1	2	3	4
Doing light domestic duties (cleaning room/table cooking/ dusting)	0	1	2	3	4
While sitting cross-legged on floor	0	1	2	3	4
Rising from cross cross-legged position	0	1	2	3	4
While squatting on the floor	0	1	2	3	4
Total Difficulty Score (Sum of Score of items 1 to 17)					
Womac Total Score (Sum of Pain+Stifness+ Difficulty Scores)					

The primary effectiveness endpoint was the mean change in the symptoms of pain as measured by the pain subscale of the WOMAC Index (Modified - CRD Pune Version) from baseline (Day 1) to Week 6 (±1 day) post-treatment with polmacoxib 2 mg capsule.

The secondary endpoints were the mean change in pain subscale of the WOMAC Index (Modified - CRD Pune Version) from baseline (Day 1) to Week 3 (±1 day). The mean change in the stiffness subscale of the WOMAC Index (Modified - CRD Pune Version), difficulty (in the Physical function) subscale of the WOMAC Index (Modified - CRD Pune Version), total WOMAC Index (Modified - CRD Pune Version) score, and severity of pain as measured by a 10-point VAS, from baseline (Day 1) to Week 3 (±1 day), and Week 6 (±1 day), post-treatment with polmacoxib 2 mg capsule. The secondary endpoints also included the global assessment of effectiveness score, as rated by the investigator and patients on a 10-point VAS (where 0 = Worsened and 10 = Significantly Improved), at Week 3 (±1 day) and Week 6 (±1 day) post-treatment with polmacoxib 2 mg capsule and time of onset of action (in minutes), in patients with knee/hip osteoarthritis, post-taking the first dose of polmacoxib 2 mg capsule on Day 1.

Safety outcomes encompassed the occurrence of adverse drug reactions (ADRs), other pharmacovigilance-relevant information (OPRI), serious ADRs and OPRI, as well as ADRs resulting in treatment discontinuation during the study. Overall tolerability was evaluated by both physicians and patients at Week 6 (±1 day).

Statistical analysis

The sample size of the study was computed to detect a meaningful improvement in the WOMAC INDEX pain subscale after six weeks of treatment. Based on a study by Lee et al., the standard deviation (SD) of the change from baseline in six weeks was estimated to be 9.5 [3]. Assuming a mean reduction of 2.5 points in the WOMAC INDEX pain subscale, the study had 85% power to detect this level of improvement at a 5% level of significance, with 130 evaluable subjects. Assuming a dropout rate of 20%, 150 patients were enrolled in the study.

The safety population included all patients who received at least one dose of study medication. Those in the safety population with at least one follow-up visit comprised the intention-to-treat (ITT) population. Patients in the ITT group who completed the study according to the protocol were included in the per-protocol (PP) population and considered for effectiveness analysis.

The continuous variables are presented as mean and SD. A paired t-test at a 5% level of significance was used to determine statistical significance in continuous variables. Categorical variables are presented as frequency (n) and percentages (%). Data was analyzed using IBM SPSS Statistics for Windows, Version 26 (Released 2020; IBM Corp., Armonk, New York, United States).

## Results

Demographics and baseline characteristics

A total of 150 patients (male: female, 89:61) with osteoarthritis were enrolled in the study. The mean (SD) age was 46.6 (9.9) years, with the majority (82.0%) diagnosed with knee osteoarthritis. All patients received oral polmacoxib 2 mg once daily and completed the study as per protocol. Demographic and baseline characteristics are detailed in Table 2.

**Table 2:** Patient demographics and baseline characteristics of the ITT population BMI: body mass index; ITT: intention-to-treat; SD: standard deviation

Parameter	Overall (N = 150)
Sex, n (%)	
Male	89 (59.3)
Females	61 (28.8)
Age (years), mean ± SD	46.6 ± 9.9
Height (cm), mean ± SD	164.9 ±1 0.3
Weight (kg), mean ± SD	67.0 ± 10.5
BMI (kg/m^2^), mean ± SD	24.7 ± 3.7
Affected joint, n (%)	
Knee	123 (82.0)
Hip	27 (18.0)

Effectiveness of once-daily polmacoxib 2 mg capsule

Significant improvement in symptoms of knee/hip osteoarthritis was reported, post six weeks of treatment with once-a-day polmacoxib 2 mg capsule. The mean (SD) WOMAC Index (Modified - CRD Pune Version) pain score declined significantly (p<.001) by -2.6 (2.1) at Visit 2 (Week 3±1 Day), and by -5.9 (3.1) at Visit 3 (Week 6±1 Day) compared to the baseline mean (SD) pain score of 11.4 (4.6).

Compared to the baseline mean (SD) stiffness score of 4.9 (2.1), the mean (SD) WOMAC Index (Modified - CRD Pune Version) stiffness score showed a significant reduction (p < 0.001) of -1.0 (1.0) at Visit 2 (Week 3 ± 1 day) and -2.4 (1.3) at Visit 3 (Week 6 ± 1 day). Similarly, the baseline mean (SD) difficulty (physical function) score of 41.6 (15.7) declined significantly (p < 0.001) by -7.8 (5.9) at Visit 2 and -19.8 (10.2) at Visit 3. Consequently, the mean (SD) total WOMAC Index (Modified - CRD Pune Version) score decreased significantly (p < 0.001) by -11.4 (7.9) at Visit 2 and -28.1 (13.8) at Visit 3, compared to the baseline score of 57.9 (21.9).

Likewise, the mean (SD) pain intensity score (VAS) decreased significantly (p < 0.001) from a baseline of 7.5 (0.9) by -1.3 (1.3) at Visit 2 and -2.9 (1.9) at Visit 3. The results of the primary and secondary effectiveness parameters are summarized in Table 3.

**Table 3:** Mean change in effectiveness parameters at Weeks 3 and 6 compared with baseline (PP population, N = 150) N: number of patients in PP set; PP: per protocol; CI: confidence interval; SD: standard deviation; VAS: Visual Analog Scale ^#^ WOMAC Index (Modified - CRD Pune Version); ^a^ Analyzed using paired sample t-test

Variables	Baseline Mean (SD)	Week 3 Mean (SD)	Mean (SD) Difference (95% CI)	t-statistic	P-value^a^	Week 6 Mean (SD)	Mean (SD) Difference (95% CI)	t-statistic	P-value^a^
Pain score - Womac Index^#^	11.4 (4.6)	8.8 (4.0)	-2.6 (2.1) (-3.0, -2.3)	-15.2	p<0.001	5.5 (2.7)	-5.9 (3.1) (-6.4, -5.4)	-23.5	p<0.001
Stiffness score - Womac Index^#^	4.9 (2.1)	3.9 (1.9)	-1.0 (1.0) (-1.2, -0.9)	-12.5	p<0.001	2.5 (1.5)	-2.4 (1.3) (-2.6, -2.2)	-22.5	p<0.001
Difficulty (physical function) score - Womac Index^#^	41.6 (15.7)	33.8 (14.6)	-7.8 (5.9) (-8.7, -6.8)	-16.2	p<0.001	21.8 (9.4)	-19.8 (10.2) (-21.4, -18.1)	-23.7	p<0.001
Total WOMAC Index^#^ score	57.9 (21.9)	46.5 (20.0)	-11.4 (7.9) (-12.7, -10.2)	-16.6	p<0.001	29.8 (13.2)	-28.1 (13.8) (-30.3, -25.8)	-24.9	p<0.001
Intensity of pain (VAS) score	7.5 (0.9)	6.2 (1.5)	-1.3 (1.3) (-1.5, -1.1)	-12.4	p<0.001	4.6 (1.7)	-2.9 (1.9) (-3.2, -2.6)	-18.8	p<0.001

**Figure 1:**
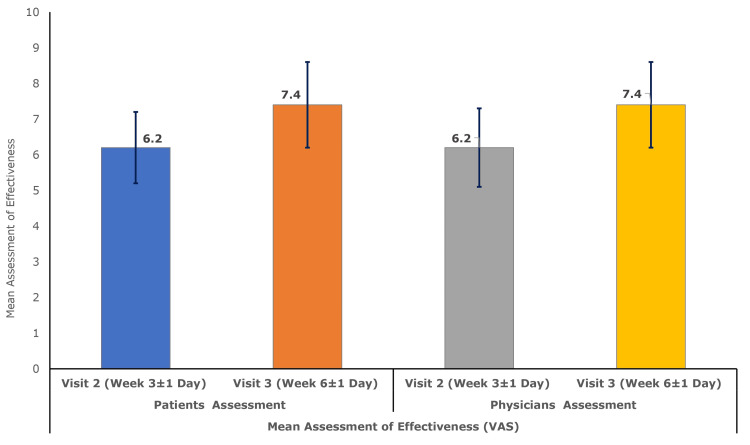
Mean (± SD) global assessment of effectiveness (VAS) by physician and patient (per-protocol population, N = 150) VAS: Visual Analog Scale

The study investigator rated the mean (SD) effectiveness of polmacoxib 2 mg capsule treatment, measured using the VAS, as 6.2 (1.1) at Visit 2 (Week 3 ± 1 day), which further improved to 7.4 (1.2) at Visit 3 (Week 6 ± 1 day). Similarly, patients rated the mean (SD) effectiveness of the once-daily polmacoxib 2 mg capsules as 6.2 (1.0) and 7.4 (1.2) at Visit 2 and Visit 3, respectively (Figure 1).

The onset of action of the study drug was reported by six patients (4.0%) within 15 minutes, 69 patients (46.0%) within 30 minutes, 62 patients (41.3%) within 45 minutes, and 13 patients (8.7%) within 60 minutes of the first dose. Compliance with the once-daily polmacoxib 2 mg capsule regimen was assessed using both patient-maintained medication diaries and pill count at each follow-up visit. Based on these methods, 98.7% of patients were classified as compliant (missed ≤5% of doses), while 1.3% were moderately compliant (missed >5% to ≤15% of doses).

Safety and tolerability

No ADRs or OPRIs were reported during the eight-week (six weeks of treatment + two-week follow-up) study period. Three (2.0%) patients reported three (2.0%) incidences of AEs. One (0.7%) incidence each of diarrhea, nasopharyngitis, and pyrexia was reported. All three incidences of AEs were mild in nature and had no relationship to the study medication. Concomitant medication was prescribed for two (1.4%) incidences of AEs, and no action was taken in one (0.7%) incidence of AEs reported. All three AEs had resolved during the eight-week study period. No severe AEs leading to discontinuation of study medication were reported.

**Figure 2:**
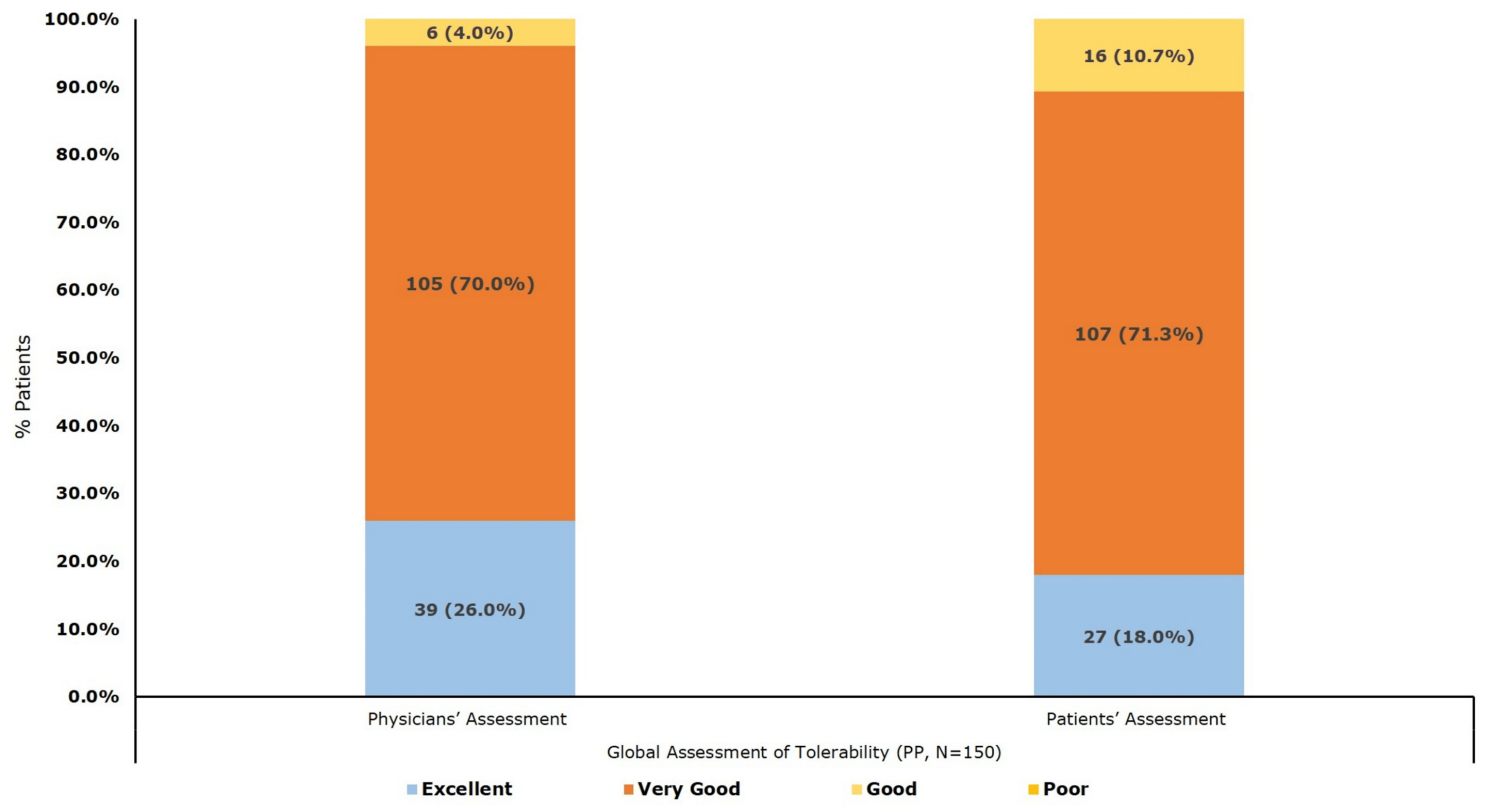
Global assessment of tolerability by physician and patient (per-protocol population, N = 150)

Overall, treatment with once-a-day polmacoxib 2 mg capsule was well-tolerated, with the study investigator reporting 'Excellent to Very Good' tolerability in 96.0% of patients and Good in 4.0% of patients. Correspondingly, 89.3% and 10.7% of patients described the tolerability of polmacoxib 2 mg capsules as "Excellent to Very Good" and "Good," respectively (Figure 2).

## Discussion

This study investigated the effectiveness, onset of action, and safety and tolerability of a new analgesic, polmacoxib. Indian patients with hip/knee osteoarthritis were given an oral dose of polmacoxib 2 mg once daily, and evaluation was done over eight weeks (six weeks of treatment plus two-week follow-up).

The effectiveness of polmacoxib was evaluated by assessing its impact on pain, stiffness, and difficulty in physical function among participants. Substantial clinical improvements were observed at Week 3, with continued symptom relief observed till Week 6. A significant reduction in pain severity was noted, as measured by the WOMAC Index (Modified - CRD Pune Version) pain subscale. The baseline mean (SD) pain score of 11.4 (4.6) declined significantly by -2.6 (2.1) at Week 3, and by -5.9 (3.1) at Week 6, reflecting marked pain relief. The findings align with previous studies, where mean WOMAC pain scores at Week 6 were reduced by -6.338 and -4.943, from the respective baseline pain scores in PP populations treated with polmacoxib [3,24]. Further, the intensity of pain assessed via the VAS also demonstrated significant reductions from a baseline mean (SD) of 7.5 (0.9), by -1.3 (1.3) at Week 3, and by -2.9 (1.9) at Week 6. Thus, the present results reinforce the reproducibility of polmacoxib’s analgesic efficacy.

Significant improvements in joint stiffness were evident, with the WOMAC Index (Modified - CRD Pune Version) stiffness subscale score decreasing from a baseline mean (SD) of 4.9 (2.1) by -1.0 (1.0) at Week 3 and -2.4 (1.3) at Week 6. This reduction in stiffness, along with the pain relief, contributed to improvement in physical function assessed by the WOMAC Index (Modified - CRD Pune Version) difficulty (in physical function) subscale score. Compared to the baseline mean (SD) difficulty (in physical function) subscale score of 41.6 (15.7), it declined by -7.8 (5.9) at Week 3 and -19.8 (10.2) at Week 6. These improvements are consistent with previous studies, reporting mean differences of -2.1 and -17.4 in WOMAC stiffness and difficulty in physical function scores, respectively, at Week 6 in the polmacoxib-treated population [3]. Additionally, both investigators and patients reported increased perceived effectiveness of polmacoxib treatment. On a VAS scale, investigator-rated mean (SD) effectiveness increased from 6.2 (1.1) at Week 3 to 7.4 (1.2) at Week 6. Similarly, patient-reported scores rose from 6.2 (1.0) to 7.4 (1.2) over the same period, further reinforcing the clinical benefit and acceptability of polmacoxib in this population.

Polmacoxib 2 mg demonstrated a rapid onset of action, with the majority of the participants reporting pain relief within 30 and 45 minutes of the first dose. Treatment adherence was notably high, with 98.7% of patients classified as fully compliant (missing ≤5% of doses). A previous study reported that ≥10% of subjects developed AEs relating to GI disorders and general disorders during polmacoxib therapy [3]. However, the current study demonstrated an acceptable safety and tolerability profile for polmacoxib 2 mg capsules. Only 2% of participants reported AEs, all of which were of mild intensity and deemed unrelated to the study medication by the investigator. Importantly, no GI or CV-related AEs were observed, which underscores the improved tolerability of polmacoxib in a real-world Indian population. This is further supported by investigator assessments, which rated tolerability as “Excellent to Very Good” in 96% of cases, and by patient-reported outcomes, where 89.3% of participants gave similar ratings.

This study has some limitations, primarily due to its single-arm, open-label design. The lack of a control or placebo group limits the ability to fully separate the effects of the treatment from natural improvement or placebo response. Additionally, the open-label setting may have introduced some bias in the reporting of subjective outcomes such as pain and stiffness. However, the study was adequately powered, and the use of validated assessment tools supports the reliability of the findings. Standardized definitions and consistent evaluation of clinical characteristics help enhance the robustness of the results. Importantly, conducting the study in a real-world clinical setting increases the relevance of findings to everyday practice. These results provide valuable preliminary evidence for the effectiveness and tolerability of polmacoxib and may help inform the design of future large-scale, long-term studies to further assess its sustained benefits and safety profile, including CV safety.

## Conclusions

This prospective, multicenter, open-label observational study indicates that six weeks of once-daily treatment with polmacoxib 2 mg led to meaningful improvements in pain, stiffness, and physical function in Indian patients with knee or hip osteoarthritis. The treatment was generally well tolerated and demonstrated an acceptable safety profile through the extended eight-week follow-up period. While the single-arm, open-label design limits the ability to attribute outcomes solely to the intervention, the use of validated outcome measures, adequate sample size, and real-world clinical settings supports the relevance of the findings. These results add to the growing body of evidence supporting the role of polmacoxib in osteoarthritis management, making it a practical addition to routine clinical care.
